# Aboriginal uses and management of ethnobotanical species in deciduous forests of Chhattisgarh state in India

**DOI:** 10.1186/1746-4269-5-20

**Published:** 2009-08-04

**Authors:** Chandra Prakash Kala

**Affiliations:** 1 Ecosystem & Environment Management, Indian Institute of Forest Management, P.B. No. 357, Nehru Nagar, Bhopal – 462 003, Madhya Pradesh, India

## Abstract

A study on the native uses of ethnobotanical species was carried out in the south Surguja district of Chhattisgarh state in India with the major objective of identifying different food and medicinal plant species and also to understand their ongoing management and conservation. Through questionnaire and personal interviews, a total of 73 ethnobotanical species used by tribal and non-tribal communities were documented, of these 36 species were used in curing different types of diseases and 22 were used as edible food plants. This rich traditional knowledge of local people has an immense potential for pharmacological studies. The outside forces, at present, were mainly blamed to change the traditional system of harvesting and management of ethnobotanical species. The destructive harvesting practices have damaged the existing populations of many ethnobotanical species *viz*., *Asparagus racemosus, Dioscorea bulbifera, Boswellia serrata, Buchnania lanzan, Sterculia urens *and *Anogeissus latifolia*. The sustainable harvesting and management issues of ethnobotanical species are discussed in view of their conservation and management.

## Background

The Chhattisgarh state of India is one of the best representatives of the Deccan Peninsular bio-geographic zone that obtains biodiversity rich deciduous forests. About 44% geographical area of Chhattisgarh state is under various types of forests with rich plant diversity, of these many species are of ethnobotanical importance. In order to conserve and maintain the natural populations of these ethnobotanical species as well as to meet their requirements, the Chhattisgarh State Minor Forest Produce Co-operative Federation has been established, which deals with various conservation, development and livelihood issues at state level. A large number of hunter-gatherer societies live in the forests of Chhattisgarh from historical times, and these tribal and non-tribal groups meet their daily requirements from the surrounding forest resources. About 44 tribal communities live in Chhattisgarh state. Over the years of trial and errors, they have accumulated a great deal of knowledge on the utility of surrounding biodiversity. This traditionally occupied knowledge is transmitted by oral means and is mostly acquired through learning-by-doing approaches [1-5].

The plant based resources form a large share on which rural communities depend for food and medicines [6]. Besides, the surrounding forest resources are used for forage, construction of dwellings, making household and agricultural implements, dyes, and for fire, shade, gums, fibers etc. Generally, the traditional knowledge on the use of plant resources is dwindling due to several reasons including shift in attitude towards a more western lifestyle and declining interest of younger generations to carry forward the tradition. The traditionally occupied ethnobotanical knowledge is mostly, at present, restricted to far-flung areas away from invasion of modern cultural forces [7]. The rural area in Surguja district of Chhattisgarh state is inhabited by many tribal groups [2], which possess a great deal of knowledge on the various plant resources. Therefore, the present study was undertaken in the southern part of Surguja district with the major objective of assessing the indigenous knowledge of rural tribal communities associated with the ethnobotanical species. Attempts were also made to understand the harvesting practices and management of ethnobotanical species.

## Methodology

### Study area

The Surguja district lies in the north of Chhattisgarh state in India between 23° 37' 25" to 24° 6' 17" north latitude and 81° 34' 40" to 84° 4' 40" east longitude. The states of Uttar Pradesh, Jharkhand, Orissa and Madhya Pradesh encircle Surguja district, and the Vindhyachal-Baghelkhand region of peninsular India overlaps the southeastern part of the Surguja. Ambikapur is the district's headquarter. The forests are dry deciduous type and primarily dominated by *Shorea robusta. Madhuca indica, Anogeissus latifolia *and *Semecarpus anacardium *are the major companion species of *Shorea robusta *found in these forests.

Mythologically, the Lord Rama had visited Surguja during his 14 years of exile into the forests. Many places of Surguja *viz*., Ramgarh, Sita-Bhengra and Laxmangarh have been associated with the epic Ramayana and named after the Lord Rama, Laxmana and Goddess Sita. Surguja is one of the important tribal regions of Chhattisgarh. The major ethnic groups in the study area were Gond, Majhwar and Baiga. Yadav, Urawoo, Dash and Chauhan were among the other rural communities in the region. Apart from forest resource collection, the villagers practice agriculture and raise some crops, such as, paddy and maize. Comparatively, agriculture is practiced largely by the Gond tribe and livestock raring is practiced by Yadav.

### Ethnobotanical exploration

Literature survey carried out on the ethnobotanical investigations reveals that there is almost no study available so far in the south Surguja region, however, some sporadic studies are available in the adjacent areas and districts [2,8-12]. Hence, the field surveys in tribal villages of south Surguja district of Chhattisgarh covering Kete, Ghatbarra, Parsa, Tara, Pendrakhi, Parogia, Hariharpur, Shivnagar, Fatepur and Bhandargaun were undertaken during July to September 2008 for gathering data on the uses of various plant species. During the survey period, information was also gathered using semi-structured questionnaires on plant parts used for food, medicine, vegetable, fibers, dyes, gums, agricultural implements and types of ailments cured by the use of plant species. Cross-checking of data was made with the help of group discussions among different age classes of tribal and non-tribal villagers that include both the genders of the society. The participant observation method was also employed to understand the methods and techniques adopted by tribal to use plant and plant parts. The surrounding forested area and agricultural land of villagers were also surveyed with local youths and knowledgeable elders for the identification of various ethnobotanical species and their indigenous uses. The nomenclature and botanical identity of the plant species follows Witt [1], Haines [13], Roy et al. [14] and Panigrahi and Murti [15].

## Results and discussion

**Figure 1:**
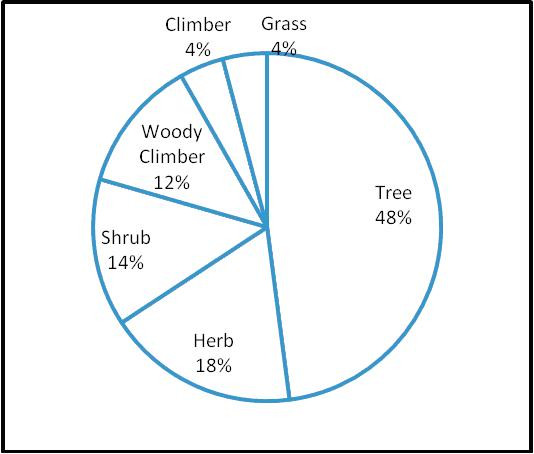
**Distribution of ethno-botanical species across various life forms in the study area**.

The present investigations have recorded 73 ethnobotanical species used by tribal and non-tribal communities in southern part of Surguja district of Chhattisgarh state in India (Table 1). The recorded ethnobotanical species were distributed over various life forms, of which 35 were tree species, 13 were herbs, 10 were shrubs, 9 were woody climber, and 3 each were grass and climber species, respectively (Figure 1). In terms of number of ethnobotanical species, Leguminoceae was the most dominant family, though, 37 plant families have their presence in the study area. These ethnobotancial species had diverse uses *viz*., medicine, beverages, vegetables, tonic, fish poison, mosquito repellent and as dying clothes. Of the total ethnobotanical species, the highest numbers of plant species (n = 36) were used in curing different types of diseases, followed by wild edible plants (n = 22). Of the remaining ethnobotanical species, 5 plant species were used as tonic, 4 as dye yielding plant, 2 for preparing beverages, 5 as fish poison, and one plant species was exclusively used as mosquito repellent.

**Table 1:** Ethnobotanical species used by local people for various purposes

**Sl. No**.	**Botanical Name**	**Local Name**	**Family**	**Habit**	**Part used**	**Uses**	
						
						**Present survey**	**Secondary sources on Surguja and adjoining areas**
1	*Acacia arabica *Willd.	Bambhur, Bamura	Mimosaceae	Tree	Bark	Liquor, dye	Bark – dye; Leaves and pod – fodder; Wood – fuelwood [1]

2	*Acacia caesia *W. et. A.	Goriyar, Garur	Mimosaceae	Woody Climber	Root	Fish poison	-

3	*Achyranthus aspera *L.	Gathiya, Aghada	Amaranthaceae	Herb	Leaf	Diuretic, tonic, antidote for insect and scorpion bite	Shoot and leaf – scorpion bite [23]

4	*Acorus calamus *L.	Bach	Acoraceae	Herb	Rhizome	Stomachache, anthelmentic	Root – fever, cough [11]; Medicine [5]*; Snake bite, liver disorder, cough and cold [23]

5	*Aegle marmelos *Correa.	Bel	Rutaceae	Tree	Fruit	Dysentery	Medicine [5]*; Fruit – dysentery; Wood – agricultural implements [1]

6	*Antidesma diandrum *Roth.	Saroti, sarwat	Euphorbiaceae	Shrub	Leaf	Vegetable	-

7	*Asparagus racemosus *Willd.	Kargi	Liliaceae	Herb	Root	Internal pain, tumors, tonic	Root – fever [23]; Medicine [5]*

8	*Bauhinia variegata *L.	Champa	Caesalpiniaceae	Tree	Bark	Dye	Piles, dysentery, leprosy [22]

9	*Bombax malabaricum *DC.	Semer, semal	Malvaceae	Tree	Flower	Edible	-

10	*Boswellia serrata *Roxb.	Saliha	Burseraceae	Tree	Resin	Arthritis	-

11	*Buchanania lanzan *Spr.	Char	Anacardiaceae	Tree	Fruit, bark	Cough, skin diseases	Bark – snake bite [23]; Medicine [5]*

12	*Butea monosperma *(Lamk.) Taub.	Parsa	Leguminoceae	Shrub	Flower, gum, seed	Diarrhea, leucorrhea, skin diseases	Diarrhea, dysentery snake bite, piles [22]; Stem – menstrual pain [23]

13	*Caesalpinia bonducella *Flem.	Gataran	Caesalpiniaceae	Woody Climber	Seed	Tonic	-

14	*Careya arborea *Roxb.	Kumahi, kumhi	Myrtaceae	Tree	Leaf, fruit	Edible	Snake bite, fever [22]

15	*Carissa spinarum *DC.	Kari	Apocynaceae	Shrub	Fruit	Edible	Fruit – edible; Thorny branches – fencing fields [1]

16	*Celastrus peniculata *Willd.	Unjain	Celastraceae	Woody Climber	Seed	Tumors	-

17	*Ceropegia bulbosa *Roxb.	Bosiya kandha	Asclepiadaceae	Climber	Tuber	Tonic, aphrodisiac	Tuber – edible [1]

18	*Chlorophytum tuberosum *(Roxb.) Baker	Safed musli	Liliaceae	Herb	Tuber	Skin diseases, tonic	Medicine [5]*

19	*Chloroxylon swietenia *DC.	Bhirra, bharahi	Meliaceae	Tree	Leaf	Mosquito repellent; for fishing	Wood – agricultural implements [1,13]

20	*Cissus quadrangularis *L.	Hathjod	Vitaceae	Climber	Stem	Antimicrobial, fracture, fertility	Medicine [5]*

21	*Cordia macleodii *H. f. & Th.	Dhahjar, Dahgan	Boraginaceae	Tree	Stem	Furniture, agricultural implements	Stem – furniture and agricultural implements [1]

22	*Cordia mixa *L.	Lasoda	Boraginaceae	Tree	Fruit	Edible	-

23	*Costus speciosus *(Koen.) Sm.	Kewu, ban haldi	Zingiberaceae	Herb	Rhizome	Fishing	Medicine [5]*

24	*Curcuma angustifolia *Roxb.	Tikhur	Zingiberaceae	Herb	Rhizome	Wounds	Medicine [5]*

25	*Cynodon dactylon *Pers.	Doob	Poaceae	Grass	Root	Liver disorder, wounds	To increase blood in the body [11]

26	*Dalbergia latifolia *Roxb.	Sirish	Leguminoceae	Tree	Timber	Agricultural implements	-

27	*Delbergia paniculata *Roxb.	Dhobnin, dhobin	Leguminoceae	Tree	Stem	Timber, for making door	-

28	*Dendrocalamus strictus *Nees	Bans	Poaceae	Grass	Stem	Basket, mat	Container for keeping tobacco, fishing net [12]

29	*Derris scandens *Benth.	Nakuwa kandha	Leguminoceae	Woody Climber	Root	Edible	-

30	*Dillenia pentagyna *Roxb.	Kurkut, korkut	Dilleniaceae	Tree	Fruit	Edible	-

31	*Dioscorea bulbifera *L.	Agitha	Dioscoraceae	Climber	Tuber	Edible	Tuber – edible, famine food [1]

32	*Dioscorea *sp	Gethi kandha/Karuha	Dioscoraceae	Herb	Fruit	Edible	-

33	*Diospyrus melanoxylon *Roxb.	Tendu	Ebenaceae	Tree	Root, fruit	Snake bite, fruit edible	Fruit – edible; timber tree [1]; Leaf – Beedi (cheap cigarette) wrapper [21]; Wood – toy for children [12]; Medicine [5]*

34	*Diospyrus montana *Roxb.	Makar tendu	Ebenaceae	Tree	Fruit	Edible	-

35	*Echinochloa colonum *(L.) Link	Sawa, sama	Poaceae	Grass	Seed	Edible	-

36	*Elaeodendron glaucum *Pers.	Mamri, Jamrasi	Celastraceae	Shrub	Root	Snake bite	Wood – comb, picture frame [1]

37	*Eugenia heyneana *Wall.	Jamti	Myrtaceae	Tree	Fruit	Edible	Fruit – edible [1]

38	*Ficus bengalensis *L.	Gad nifir	Moraceae	Tree	Latex, bark	Dysentery	-

39	*Ficus infectoria *Roxb.	Pakri, Pakhri	Moraceae	Tree	Leaf	Vegetable	-

40	*Flacourtia indica *(Burm. f.) Merr.	Ramkatayi, kaker	Bixaceae	Shrub	Fruit	Edible	Root – skin diseases [22]

41	*Gardenia latifolia *Ait.	Paprol, piprol, Mali	Rubiaceae	Tree	Fruit	Perfume	-

42	*Garura pinnata *Roxb.	Khenkara, Kekad, Kenkar	Burseraceae	Tree	Bark, fruit	Snake bite in water, wounds, fruit edible, pickle	Fruit – fodder for cattle [11]; Bark- diabetes [22]

43	*Gloriosa superba *L.	Kharha godi, karihari	Liliaceae	Herb	Root	Tumor	-

44	*Helicterus isora *L.	Aaithi, marorphali	Sterculiaceae	Shrub	Bark, fruit	Colic, intestinal disorder, used to make rope	Medicine [5]*

45	*Hibiscus abelmoschus *L.	Kapalsiya kandha	Malvaceae	Herb	Root	Blood in urine	Medicine [5]*

46	*Holarrhena antidysenterica *Wall.	Koriya	Apocynaceae	Tree	Root, bark	Fever, dysentery	Bark – asthma [22]

47	*Indigofera pulchella *Roxb.	Bhul bhuli, Ghirhul	Leguminoceae	Shrub	Flower	Edible vegetable	-

48	*Ipomoea mauritiana *Jacq.	Patal kohra	Convolvulaceae	Woody Climber	Root	Digestion	Medicine [5]*

49	*Madhuca indica *Gmel	Mahuwa	Sapotaceae	Tree	Fruit, root, flower, seed	Liquor; barks use in bleeding gums, ulcers and diabetes	Seeds oil – snake bite, scorpion bite [23]

50	*Mangifera indica *L.	Aam	Anacardiaceae	Tree	Fruit	Edible	Bark – children bath for health [11]; Wood – toy for children [12]

51	*Murraya koenigii *Spreng.	Mithi neem	Rutaceae	Shrub	Leaf, fruit	Edible	-

52	*Ougenia dalbergioides *Benth.	Sandhan, tilsa	Leguminoceae	Tree	Stem, bark	Furniture, intoxicate fish	-

53	*Peucedanum nagpurense *(Cl.) Prain	Tejraj	Asteracease	Herb		Medicine	Root – to increase semen [11]

54	*Phoenix acaulis *Buch	Chind	Palmaceae	Herb	Fruit	After child birth, fruit edible	Leaves – local umbrella [12]

55	*Phyllanthus emblica *L.	Awala	Euphorbiaceae	Tree	Fruit	Edible	Medicine [5]*

56	*Pterocarpus marsupium *Roxb.	Bija	Leguminoceae	Tree	Wood	Timber, for making door	Leaves – fodder; Wood – bullock cart and cots [11]; Gum – toothache [23]

57	*Punica granatum *L.	Anar	Lythraceae	Tree	Fruit	Edible	-

58	*Randia dumetorum *Lamk.	Menda, Mainfal	Rubiaceae	Tree	Fruit	Fish poison	Unripe fruit – fish poison; Bark – medicine* [1]

59	*Ricinus communis *L.	Arandi	Euphorbiaceae	Shrub	Seed, root	Dandruff, skin diseases, epilepsy	-

60	*Semecarpus anacardium *L.	Bhelwa	Anacardiaceae	Tree	Fruit	Edible, oil is massaged on infected parts of the body	-

61	*Shorea robusta *Gaertn.	Sal, sarayi	Dipterocarpaceae	Tree	Stem, resin	Timber, resin for fire and spasm	Seed – edible oil [21]; Medicine [5]*; Timber [1]

62	*Soymida fabrifuga *A. Juss.	Rohina, rohan	Meliaceae	Tree	Bark	Muscular pain	-

63	*Symplocos racemosa *Roxb.	Lodh	Symplocaceae	Tree	Bark	Dye	Medicine [5]*

64	*Terminalia arjuna *Bedd.	Kahua, arjun	Combretaceae	Tree	Bark	Medicine	Bark – fever, high blood pressure [11]

65	*Terminalia bellerica *Roxb.	Baira	Combretaceae	Tree	Fruit	Cough	Medicine [5]*

66	*Terminalia chebula *Retz.	Harra	Combretaceae	Tree	Fruit	Cough	Fruit – cough, asthma, black dye [11]; Indigestion [21]; Medicine [5]*; Seeds – wounds [23]

67	*Thespesia lampus *Dalz.	Masbandi, mundi	Malvaceae	Herb	Young twig	Fiber; rope	-

68	*Urginea indica *Kunth.	Ban pyaz	Hyacinthaceae	Herb	Tuber	Scorpion bite	Medicine [5]*

69	*Vallaris heynei *Spr.	Dudhiya kandha	Apocynaceae	Woody Climber	Latex	Lactating mother, cow, buffalo	-

70	*Ventilago madraspatana *Gaertn.	Kyonti, Keoti	Rhamnaceae	Woody Climber	Root bark	Chocolate & red dye	Bark – rope fiber [1]

71	*Vitis carnosa *Wall.	Dhokar bela	Vitaceae	Woody Climber	Root	Bodyache, drink	Root and leaf – boils, tumor [1]

72	*Woodfordia floribunda *Salisb.	Dhai, Dhawai	Lythraceae	Shrub	Flower	Red dye	Medicine [5]*

73	*Zizyphus rugosa *Lamk.	Churaban, Churna	Rhamnaceae	Woody Climber	Whole	Bodyache	-

* Particular use is not mentioned.

### Medicinal Plants

The total documented medicinal plant species were distributed over 26 families and have occupied various life forms, of which 14 were herbaceous species (3 climbers, 1 grass and 10 forbs), 13 were tree species, 6 were woody climber species and 3 were shrub species (Table 1). Combretaceae and Liliaceae had the highest number of species used in curing diseases. Different plant parts of these species, such as, root, tuber, leaf, fruit, bark, resin, seed and latex were used as medicine. In majority of cases, root (14 species) was used for preparing medicine, followed by fruit (7 species) and bark (5 species). More than one plant parts of 4 plant species *viz*., Garura pinnata Roxb., Helicterus isora L., Holarrhena antidysenterica Wall. and Ficus bengalensis L. were used as medicine. Cough, bodyache, dysentery, cut-wounds, scorpion bite, snake bite, muscular pain, and indigestion were among the ailments cured by using these plant species.

Many species of snakes including cobra were found in the study area, and snakebite was one of the frequent problems. In case of snakebite, the person was treated by some specialized expert, who used some plant species and also chanted some spiritual words while curing snakebite. *Diospyrus melanoxylon *Roxb., *Elaeodendron glaucum *Pers., and *Garura pinnata *were some of the important plant species used for curing snakebite. Similarly, the scorpion bite was treated by using the leaf paste of *Achyranthus aspera *L., and tuber of *Urginea indica *Kunth. The Surguja district is a mosquito prone area, and death by malarial fever is a common phenomena. The local people spend most of the time in the forest for rearing of their livestock, collection of fuelwood, fodder, medicinal and edible plants. To keep mosquito away from their body, they rubbed leaves of *Chloroxylon swietenia *DC. on the exposed body parts and also put its twigs on the head and back.

The study area harboured several important medicinal plants used in Ayurvedic medicine. *Terminalia arjuna *Bedd., *Terminalia bellerica *Roxb., *Terminalia chebula *Retz., *Phyllanthus emblica *L., *Holarrhena antidysenterica *Wall., *Hibiscus abelmoschus *L., *Gloriosa superba *L., *Dioscorea bulbifera *L., *Aegle marmelos *Correa., *Boswellia serrata *Roxb., *Acorus calamus *L. and *Asparagus racemosus *Willd. were among the prominent ingredients of the Ayurvedic medicine. All three species viz., *Terminalia arjuna*, *Terminalia bellerica *and *Phyllanthus emblica *of important Ayurvedic medicine 'Triphala' were available and used by local people in the study area.

### Wild edible plants

Wild edible plants were one of the prime sources of livelihood to the rural communities of Surguja district. Various plant parts *viz*., fruit, leaf, flower, tuber, rhizome, root and seed were source of food to the residents of the study area. Of total 22 wild edible plant species, fruits of highest number of plant species (n = 13) were eaten as raw or after cooking by the local people. The fruits of *Carissa spinarum *DC., *Cordia mixa *L., *Phyllanthus emblica *L., *Punica granatum *L., *Diospyrus melanoxylon *Roxb., and *Flacourtia indica *(Burm. f.) Merr were consumed as food by the local people. The wild plants were also used as vegetables and the leaves and flowers of 7 plant species *viz., Antidesma diandrum *Roth., *Ficus infectoria *Roxb., and *Indigofera pulchella *Roxb. were eaten as vegetables after cooking. The roots and tubers of 3 species including *Derris scandens *Benth., and *Dioscorea bulbifera *L., were also used as food plants by the local people.

### Other ethnobotanical species

Apart from food and medicine, the consumption of locally made beverages was a common practice of most of the villagers in the study area. Majority of households used fruits and flowers of *Madhuca indica *for preparing liquor. Apart from *Madhuca indica*, some wild plant species, such as, *Acacia arabica *Willd. was used in preparing beverages. Fishing was another source of livelihoods, and for this purpose they had discovered many plant species as a fish poison, which they spread in the ponds after crushing to powder. *Acacia caesia *W. et. A., *Chloroxylon swietenia, Costus speciosus *(Koen.) Sm., *Ougenia dalbergioides *Benth., and *Randia dumatorum *Lamk., were used as fish poison by the local people.

The tribal people in Chhattisgarh have rich plant-based ethno-veterinary knowledge. About 17 species of plants used as ethno-veterinary drugs are reported from nearby districts of the study area by Shukla *et al*. [10]. Besides, the local people have also occupied knowledge on the dye yielding plants for extraction of multiple colours. The bark of *Bauhinia variegata *L. and *Symplocos racemosa *Roxb. was used to extract colours for dyeing cloths. The flower of *Woodfordia floribunda *Salisb. was used for preparing red dye. A study conducted on dye yielding plants has reported that mostly bark is used for extraction of dyes, followed by leaves and flowers [16]. The natural dyes, as used by villagers, are eco-friendly and do not impinge negative impacts like synthetic dyes [17,18]. Encouraging local people for establishing processing units of natural dyes may serve the purpose of ecosystem and environment management through avoiding the use of hazardous synthetic products.

### Harvesting practices and management issues

Traditionally, the harvesting of ethnobotanically useful species was done by the communities for their own use or nourishment. Now, the invasion of market forces has made to commercialize the important ethnobotanical species that has changed the attitude of local people towards cash and high income generation. This has subsequently led to the overexploitation of many important ethnobotanical species. The traditional collection practices of wild plants are no more viable to meet their increasing demand, at present. Due to faulty harvesting practices, such as, plucking of entire twigs in most of the cases for gathering fruits, there has been 60% damage of *Phyllanthus emblica's *trees in the nearby district of Surguja namely Bilaspur [19]. The early harvesting of rhizomes of Costus speciosus (Koen.) Sm. results in scanty seed formation and thus creates problem for its regeneration. Seed collection practice of Celastrus peniculata Willd. has affected the regeneration of this species in the wild [20]. Celastrus peniculata does not grow easily and thus there is a difficulty in propagation and cultivation of this species at large scale.

### Minor Forest Produce Co-Operative Federation

Chhattisgarh State Minor Forest Produce Co-operative Federation (CSMFPCF) is an apex organization dealing with state level policy formulation on conservation, collection, value addition and marketing of non-wood forest produce. CSMFPCF is involved in the collection and sale of the forest produce (i.e., Tendu leaves, Sal seed, Harra, Gums, etc.) with the help of 32 District Unions, 913 Primary Forest Produce Co-operative Societies and 10,000 Collection Centres established across the state [21]. In order to collect the forest produce, the area of collection is divided into different units and sells through tenders and auctions by the Federation. The funds for various operations are made available to Primary Societies through District Unions. The forest produce is sold by the local people/collectors to the Primary Society at every collection centre, which is responsible for transport and storage of forest produce in his godowns or the godowns of Forest Department/Federation. Primary Co-operative Societies calculate the amount of profit earned by the trade of concerned forest produce, of which 70% is provided to the collectors as wage incentives, 15% of profit each to the village resource development and development of forest/forest produce.

## Conclusion

The livelihood of local people in south Surguja of Chhattisgarh is primarily depends on the forest resources, and they use to collect many ethnobotanical species for their day-to-day activities. The wild medicinal and food plant species, as documented in the present study with the help of local people, may be screened and standardized as per scientific norms for medicinal potency and nutritive values for their wider acceptability. The need of hour is to identify and disseminate the valuable information about the important ethnobotanical species and knowledge for the benefit of society and science. To mitigate the ongoing pressures on ethnobotanical species beyond their sustainable capacity, the local people may be educated on the future consequences of nature resource over-exploitation process and environmental degradation. The forest dwellers may also be provided the sufficient resources for their basic requirements. Many ethnobotanical species provide food and medicine to the rural people [22,23]; therefore such species may be protected through non-destructive way of harvesting. Rather than exporting important ethnobotanical species in raw forms, attempts should be made at the village level to promote their processing and value addition. Agro-techniques of demand driven ethnobotanical species need to be developed and the useful tree species may be planted as agro-forestry species. Besides, the state Government and CSMFPCF may encourage agro-forestry, farm forestry and on-farm cultivation of ethnobotanically useful species.

## Competing interests

The author declares that they have no competing interests.

## References

[B1] Witt DO (1916). Descriptive List of Trees, Shrubs, Climbers and Economic Herbs of the Northern and Berar Forest Circles Central Provinces.

[B2] Tirkey A (2004). Some ethnobotanical plant species of Chhattisgarh state. Ethnobotany.

[B3] Kala CP (2005). Indigenous uses, population density, and conservation of threatened medicinal plants in protected areas of the Indian Himalayas. Conservation Biology.

[B4] Kala CP (2006). Ethnobotany and ethnoconservation of *Aegle marmelos *(L.) Correa. Indian Journal of Traditional Knowledge.

[B5] Kumar K (2007). Working Plan of North Surguja, Ambikapur. Forest Department: Government of Chhattisgarh.

[B6] Kala CP (2005). Current status of medicinal plants used by traditional *Vaidyas *in Uttaranchal state of India. Ethnobotany Research and Applications.

[B7] Kala CP (2007). Local preferences of ethnobotanical species in the Indian Himalaya: Implications for environmental conservation. Current Science.

[B8] Brijlal (1993). Ethnobotany of Baigas of Madhya Pradesh – A preliminary report. Arunachal Forest News.

[B9] Verma P, Khan AA, Singh KK (1995). Traditional phytotherapy among the Baiga tribe of Shahdol district of Madhya Pradesh, India. Ethnobotany.

[B10] Shukla AN, Singh KP, Kumar A (2007). Ethnoveterinary uses of plants from Achanakmar – Amarkantak Biosphere Reserve of Madhya Pradesh and Chhattisgarh. Journal of Non-Timber Forest Products.

[B11] Kumar V, Jain SK (1998). A contribution to ethnobotany of Surguja district in Madhya Pradesh, India. Ethnobotany.

[B12] Kumar V (1999). Some indigenous tools of Surguja district, Madhya Pradesh, India. Ethnobotany.

[B13] Haines HH (1916). Descriptive list of trees, shrubs and economic herbs of the southern circle central provinces.

[B14] Roy GP, Shukla BK, Datta B (1992). Flora of Madhya Pradesh.

[B15] Panigrahi G, Murti SK (1989). Flora of Bilaspur.

[B16] Tiwari SC, Bharat A (1998). Natural dye yielding plants and indigenous knowledge of dye preparation in Achanakmar-Amarkantak Biosphere Reserve, Central India. Natural Product Radiance.

[B17] Kala CP (2002). Indigenous knowledge of Bhotiya tribal community on wool dyeing and its present status in the Garhwal Himalaya, India. Current Science.

[B18] Mahanta D, Tiwari SC (2005). Natural dye-yielding plants and indigenous knowledge on dye preparation in Arunachal Pradesh, northern India. Current Science.

[B19] Tiwari KP (1995). Collection of Aonla (*Emblica officinalis*) fruits from forest – An impact assessment. Vaniki Sandesh.

[B20] Ved DK, Kinhal GA, Ravikumar K, Karnat M, Vijay Sankar R, Indresha JH (2003). Threat assessment and management prioritization for the medicinal plants of Chhattisgarh and Madhya Pradesh.

[B21] Chhattisgarh State Minor Forest Produce Co-operative Federation Limited; Raipur, Chhattisgarh. http://www.cgmfpfed.org/forestproduce1.htm.

[B22] Jain SK, Sinha BK, Gupta RC (1991). Notable plants in ethnomedicine of India.

[B23] Rai R, Nath R (2005). Use of medicinal plants by traditional herbal healers in Central India. Indian Forester.

